# Bulbourethral sling in male incontinence

**DOI:** 10.4103/0970-1591.30263

**Published:** 2007

**Authors:** Alan Shindel

**Affiliations:** Department of Surgery, Division of Urology, Washington University, St. Louis, MO, USA. E-mail: shindela@wudosis.wustl.edu

The authors report on six men who underwent treatment with a prolene mesh sling for severe stress urinary incontinence (SUI) after prostate surgery. Five of these patients had undergone transurethral resection of the prostate and one had undergone suprapubic prostatectomy. The authors report socially acceptable continence in five patients, with the other patient developing urinary retention requiring self-catheterization. These results are in agreement with a Scandinavian study which utilized a polypropylene tape placed around the urethra, with good restoration of continence.[1]

Urethral slings have become a mainstay in the treatment of female SUI. The prevailing theory on normal mechanism of female urinary continence is the ‘hammock hypothesis,’ which posits that continence in the female patient relies on the periurethral tissues to form a base on which the urethra may rest.[2] SUI is secondary to laxity of these tissues; and by recreating this base via a sling, continence may be restored.

In men there is no continence-producing ‘hammock’ to be recreated by a sling. This difference in anatomy explains why urethral slings for incontinence have been so successful in women but have not had such resounding success in men. The therapeutic goal of sling placement in men is to create a degree of urethral obstruction. Generating urethral obstruction puts the patient at risk for urinary retention, as occurred in one patient in this series.

Other therapies for male SUI include collagen injection and artificial urinary sphincter (AUS) placement. Retrograde injection of collagen at the bladder neck is safe and simple but the need for repeat treatment is high.[3] AUS provides good results in the treatment of male SUI but complications such as erosion and infection are not uncommon and the cost is prohibitive for many patients.[4]

The procedure appears to be a viable alternative to AUS and collagen injection. Follow-up for a longer term and greater numbers of patients will help the authors refine their technique and clearly determine the ultimate role of this procedure in the treatment of SUI in men after prostate surgery.
